# The Structure‐function remodeling in rabbit hearts of myocardial infarction

**DOI:** 10.14814/phy2.13311

**Published:** 2017-06-22

**Authors:** Haotian Wu, Li Li, Pei Niu, Xu Huang, Jinyi Liu, Fengshun Zhang, Wenzeng Shen, Wenchang Tan, Yiling Wu, Yunlong Huo

**Affiliations:** ^1^ School of Basic Medical SciencesNanjing University of Traditional Chinese MedicineNanjingChina; ^2^ Hebei Yiling Pharmaceutical Research InstituteShijiazhuangChina; ^3^ Department of Mechanics and Engineering ScienceCollege of EngineeringPeking UniversityBeijingChina; ^4^ College of MedicineHebei UniversityBaodingChina; ^5^ Shenzhen Graduate SchoolPeking UniversityShenzhenChina; ^6^ PKU‐HKUST Shenzhen‐Hongkong InstitutionShenzhenChina; ^7^ Key LaboratoryState Administration of Traditional Chinese Medicine (Cardiovascular and cerebrovascular collateral diseases)ShijiazhuangChina; ^8^ Hebei Province Key Laboratory of Collateral DiseasesShijiazhuangChina

**Keywords:** Coronary arterial trees, ischemic heart failure, myocardial infarction, rabbit model, structure‐function remodeling

## Abstract

Animal models are of importance to investigate basic mechanisms for ischemic heart failure (HF). The objective of the study was to create a rabbit model through multiple coronary artery ligations to investigate the postoperative structure‐function remodeling of the left ventricle (LV) and coronary arterial trees. Here, we hypothesize that the interplay of the degenerated coronary vasculature and increased ventricle wall stress relevant to cardiac fibrosis in vicinity of myocardial infarction (MI) precipitates the incidence and progression of ischemic HF. Echocardiographic measurements showed an approximately monotonic drop of fractional shortening and ejection fraction from 40% and 73% down to 28% and 58% as well as persistent enlargement of LV cavity and slight mitral regurgitation at postoperative 12 weeks. Micro‐CT and histological measurements showed that coronary vascular rarefaction and cardiac fibrosis relevant to inflammation occurred concurrently in vicinity of MI at postoperative 12 weeks albeit there was compensatory vascular growth at postoperative 6 weeks. These findings validate the proposed rabbit model and prove the hypothesis. The post‐MI rabbit model can serve as a reference to test various drugs for treatment of ischemic HF.

## Introduction

Heart failure (HF) is caused by cardiovascular diseases, for example, myocardial ischemia and infarction, hypertension, valvular heart diseases, etc (Hunt et al. 2005). The present etiology of HF is mainly associated with myocardial ischemia or infarction due to coronary artery diseases (Hunt et al. 2005). Ischemic HF occurs in more than 60% HF patients and leads to a high global burden (Moran et al. 2014). The compensatory remodeling in the left ventricle (LV) of myocardial infarction (MI) progresses into the decompensated ischemic HF over time (Lee et al. 2016). Given multiple pathophysiological alterations, the understanding of basic mechanisms for progression of ischemic HF is still rudimentary despite recent advances of relevant studies (Adams et al. 1996; Hunt et al. 2005; Monnet and Chachques 2005; Canty and Suzuki 2012; Houser et al. 2012; Isorni et al. 2015; Mohammed et al. 2015; Camacho et al. 2016).

Various animal models have been developed to investigate different stages of ischemic HF (Hasenfuss 1998; Hearse and Sutherland 2000; Muders and Elsner 2000; Monnet and Chachques 2005; Patten and Hall‐Porter 2009; Schmitto et al. 2011; Huo and Kassab 2015; Camacho et al. 2016). Since rabbit models are less expensive than large animal models, show similarities to the human heart, and allow the sequential evaluation of hemodynamic parameters and organ function (Ezzaher et al. 1992; Muders and Elsner 2000), myocardial infarction induced by a distal ligation of left circumflex artery (LCx) in rabbits has become the gold standard in the study of ischemic HF (Mahaffey et al. 1995; White et al. 2000; Edwards et al. 2002; Ziv et al. 2012). The postmyocardial infarction (post‐MI) rabbit model develops a region of complete akinesis only near the apical region of LV and hence limits the study of HF resulting from myocardial infarction on the anterior and lateral walls of the LV.

The objective of the study is to investigate the structure‐function remodeling of the LV and coronary arterial trees in rabbit hearts for 12 weeks after myocardial infarction through multiple coronary artery ligations. Here, we hypothesize that cardiac fibrotic remodeling relevant to inflammation impairs the myocardium and coronary vasculature in vicinity of myocardial infarction, which is a critical risk factor to precipitate the incidence and progression of ischemic HF. To verify the hypothesis, myocardial infarction was created in male New Zealand White rabbits through multiple ligations of coronary arteries. The follow‐up study was carried out for 12 weeks. Echocardiographic measurements were performed weekly. Hematological and hemodynamic parameters were measured before the termination. Morphometric data of coronary arterioles were obtained from micro‐CT (*μ*CT) images. Histological data were used for evaluation of myocardial infarction and fibrosis. The significance, implication, and limitation of the study were discussed in relation to potential improvements of cardiac performance in post‐MI patients.

## Methods

### Study design

Studies were performed in forty male New Zealand White rabbits weighing 2.5–3.0 kg. The experimental protocol consisted of two groups of rabbits: 20 sham animals (i.e., sham group) and 20 animals of MI through multiple coronary artery ligations (i.e., ligation group). Experiments were performed in the two groups for 6 (20 animals: 10 in each group) or 12 (20 animals: 10 in each group) weeks postoperatively. All animal experiments were performed in accordance with Chinese National and Hebei University ethical guidelines regarding the use of animals in research, consistent with the NIH guidelines (Guide for the care and use of laboratory animals) on the protection of animals used for scientific purposes. The experimental protocols were approved by the Animal Care and Use Committee of Hebei University, China.

### Animal preparation for multiple coronary artery ligations

In sterile environment, rabbits were first anesthetized by an intravenous (IV) injection of 25% urethane (4 mL/Kg) through an ear vein. Animals were then intubated and ventilated with room air using a Harvard ventilator (Inspira). Intravenous access was established for drug injection and ECG signals were monitored during the entire surgical operation.

**Figure 1:**
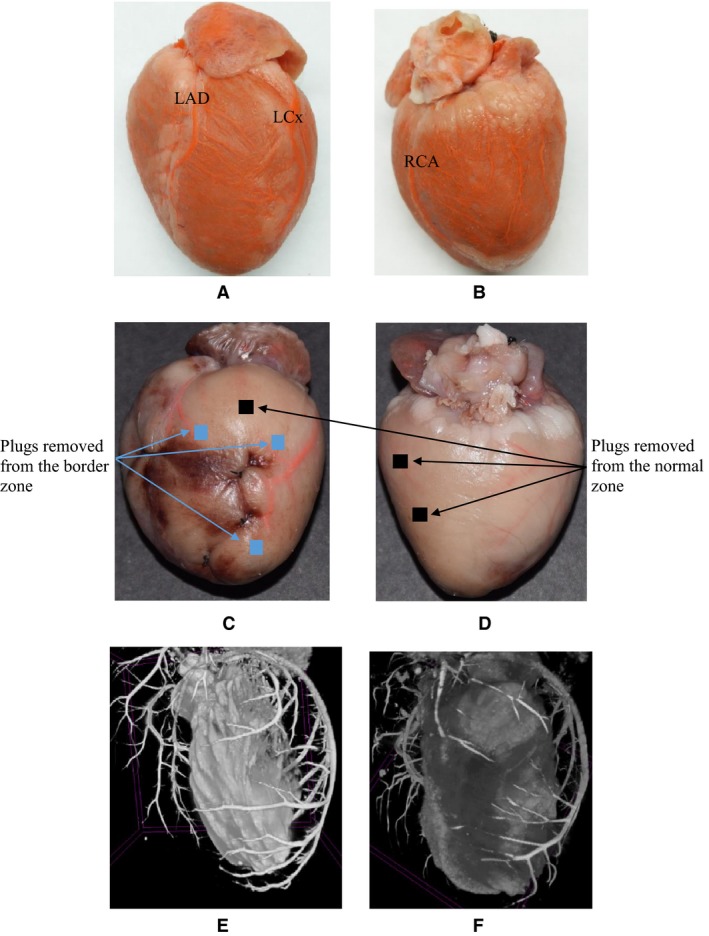
(A–B) A sham rabbit heart perfused with the casting solution (A: anterior view; B: posterior view); (C–D) A rabbit heart with four patchy areas of myocardial infarction perfused with the casting solution (C: anterior view; D: posterior view), where blue and black square regions refer to the locations of plugs (~4 × 4 mm) of myocardial tissues removed from border and normal zones, respectively; and (E–F) The LV cavity and coronary arterial trees reconstructed from *μ* CT images of (E) a sham rabbit and (F) a rabbit of myocardial infarction.

After the chest was shaved and sterilized, a left thoracotomy was performed between the third and fourth intercostal spaces, followed by a pericardiotomy (Mahaffey et al. 1995). Four suture ligations were performed transmurally on four vessel segments of the LV including the left anterior descending (LAD) artery and three primary branches of the LCx arterial tree, which created multiple, patchy areas of myocardial infarction on the anterior and apical locations of the free wall, as shown in Figure 1. Alternatively, the suture was placed but removed in sham‐operated rabbits. Lidocaine (1 mg/kg IV) was administered during the entire surgical operation. After the chest was closed, animals were intramuscularly administered a dose of Bicillin (300 000 U) for antibiotic prophylaxis and allowed to recover from the surgery. Animals were given an intramuscular injection of Bicillin (300 000 U) for 3 days.

### Echocardiographic measurements

Echocardiographic measurements were carried out weekly in rabbit hearts after myocardial infarction as well as age‐matched shams. Similar to a previous study (Fontes‐Sousa et al. 2006), M‐mode measurements of the LV, left atrium, and aorta as well as pulsed Doppler measurements of aortic outflow and mitral inflow velocities were recorded in anesthetized rabbits. The images were obtained at 12 MHz using a 2SS‐D Cardiac Probe operated by a GE Vivid E9 Color Doppler Ultrasound Scanner (GE Health). From the right parasternal short‐axis view, M‐mode tracings were made at the midventricular level. Morphometric parameters including interventricular septum end‐diastolic wall thickness (IVS_d_), interventricular septum end‐systolic wall thickness (IVS_s_), left ventricle end‐diastolic diameter (LVD_d_), left ventricle end‐systolic diameter (LVD_s_), left ventricle end‐diastolic posterior wall thickness (LVPW_d_) and left ventricle end‐systolic posterior wall thickness (LVPW_s_) were measured according to the American Society of Echocardiography leading edge rule (Sahn et al. 1978). These parameters were averaged based on five measurements. Moreover, FS (%) and EF (%) were calculated from the measured parameters as: $\frac{\left(\mathrm{LV}D_{d}-\mathrm{LV}D_{s}\right)}{\mathrm{LV}D_{d}}\times 100\%$ and $\frac{\left({\mathrm{LVD}}_{d}^{3}-{\mathrm{LVD}}_{s}^{3}\right)}{{\mathrm{LVD}}_{d}^{3}}\times 100\%$, respectively, using the software of GE Vivid E9 Color Doppler Ultrasound Scanner. From the subcostal apical four‐ and five‐chamber views, pulsed Doppler tracings were performed to determine hemodynamic parameters of mitral inflow peak velocity (MPV) and aortic outflow peak velocity (APV).

### Hematological exams

Hematological exams were demonstrated in 20 animals (10 in each group) for 11 weeks after the surgery. Standard hematological parameters including white blood cells (WBC), red blood cells (RBC), monocytes percentage (MO%), eosinophils percentage (E%), basophils percentage (B%), platelet distribution width (PDW), mean platelet volume (MVP), plateletcrit (PCT), nucleated red blood cells percentage (NRBC%), hemoglobin content (HGB), mean corpuscular volume (MCV), neutrophils percentage (NE%), lymphocyte percentage (LY%), hematocrit (HCT), and red blood cell distribution width (RDW) were determined using a LH 750 analyzer (Beckman Coulter).

### Hemodynamic measurements before the termination

Rabbits were anesthetized, intubated, and ventilated with room air for 6 or 12 weeks postoperatively. A 5F micromanometer‐tipped catheter (Millar Instruments) was inserted through the right carotid artery into the LV to record pressure waves in 30 cardiac cycles, which was repeated three times. The zero‐pressure baseline of the catheter was calibrated in 37°C saline. A conductance catheter (Suzhou Runxin Company) was also inserted to the LV to measure the volume (Huo et al. 2009). These catheters were monitored with a BIOPAC MP150. Heart rate (HR), LV systolic pressure (LVSP), LV end‐diastolic pressure (LVEDP), and rate of maximum positive and negative left ventricular pressure development ($\pm \frac{dp}{dt}$) were determined from the measured pressure waves (Pennock et al. 1997).

### Postmortem μCT measurements and 3D reconstruction

Ten rabbits in each group (they were divided into halves and terminated at postoperative 6 and 12 weeks separately) were selected for the *μ*CT analysis. A midline sternotomy was performed immediately after the hemodynamic measurements. Similar to previous studies (Chen et al. 2015; Huo and Kassab 2015), rabbits were heparinized with undiluted heparin (3 mL, 1000 USPU/mL) and then terminated with a bolus injection of saturated KCl through the jugular vein. Heparinized PBS (1 unit heparin/mL) at 37°C was injected into the thoracic aorta and drained from the sectioned inferior vena cava for 10 min. The heart was exposed and drip of heparinized PBS was used to maintain epicardial moisture. The thoracic aorta was then filled with the acetone solution at a constant pressure of 100 mmHg. After 3 min, the thoracic aorta was perfused with the casting solution (7 g Acrylonitrile Butadiene Styrene‐ABS and 10 g lead tetroxide microspheres in 100 mL acetone solution) at a constant pressure of 100 mmHg. The flow of cast solution was observed to pass by epicardial coronary arteries down to intramyocardial vessels, but it was not found in veins because lead tetroxide microspheres with diameter of 8–15 *μ*m blocked distal small arteries. The flow of cast solution was zero in 90 min prior to hardening of cast at a constant pressure of 100 mmHg. The isolated heart was stored in 10% formalin in a refrigerator for 24 h. The reperfusion was then performed to prevent the possible cast shrinkage at a constant pressure of 100 mmHg. The heart was stored in 10% formalin in the refrigerator until *μ*CT scans.

The scanning for an entire heart was performed using a Quantum GX *μ*CT scanner (PerkinElmer). Images were collected in a wide field of view (FOV = 72 mm). The X‐ray energy power and voltage were 8 W and 90 kV, respectively. The isotropic voxel size was 9 *μ*m. Total scan time for an entire heart was about 1 h. A region of myocardial infarction was identified as the distal region to a ligation with gray color and thin wall thickness. Some plugs (~4 × 4 mm) in normal and border zones (see the subsection “data analysis” for definitions of normal and border zones) were transmurally sectioned from the heart, as shown in Figure 1. The scanning for a plug was carried out similar to that for the whole heart. Total scan time for a plug was about 15 min.

Morphometric data of coronary arterial trees were extracted from *μ*CT images using a gray‐scale threshold method in a commercial software (Living Imaging 4.5.2, PerkinElmer), as shown in Figure 1. Similar to a previous study (Chen et al. 2015), low CT‐threshold was selected to include small vessel segments. The blurring of small vessel edges was corrected to yield D_correct_ by fitting a Gaussian distribution function (i.e., the modulation transfer function of *μ*CT scanner) to the line profiles followed by computation of the input square wave. To reduce the sampling error of finite discrete grid, coronary arterial trees with vessel diameter ≥36 *μ*m (four times the voxel size) were used. Moreover, vessel densities (vessel number per mm^3^) were determined in each plug using the Living Image Software.

### Histological evaluation of myocardial infarction

Ten rabbits in each group (they were divided into halves and terminated at postoperative 6 and 12 weeks separately) were selected for the histological analysis. After a midline sternotomy, rabbits were terminated with a bolus injection of saturated KCl through the jugular vein. The heart was rapidly removed and rinsed in 0.9% saline solution. Multiple plugs of myocardial tissues (~ 4 × 4 mm) were removed from the LV apex, fixed in 10% buffered formalin, and embedded in paraffin. Plugs were completely sectioned transmurally into 5 *μ*m thickness, deparaffinized with xylene, and rehydrated. The Hematoxylin/Eosin and Masson trichrome staining was performed according to standard procedures. Furthermore, infarct and fibrotic area ratios (i.e., $\frac{\text{Infarct area}}{\text{Total area}}$ and $\frac{\text{Fibrotic area}}{\text{Total area}}$) were determined using the Image‐Pro‐Plus 6.0 software (Media Cybernetics, Inc.).

### Data analysis

Morphometric and hemodynamic data were expressed as mean ± SD (standard deviation) in sham and ligation groups. Similar to a previous study (Lee et al. 2011), the LV were classified in three regimes: (1) infarct zone (i.e., a region distal to a ligation with gray color and thin wall of complete akinesis); (2) normal zone (normal work zone far from the infarct zone); and (3) border zone (i.e., transitional region of 5 mm width from the infarct zone to normal work zone). A two‐way ANOVA (SigmaStat 3.5) was used to detect the statistical difference of morphometric and hemodynamic parameters between sham and ligation groups, where *P* < 0.05 was indicative of a significant difference between the two populations.

## Results

**Figure 2:**
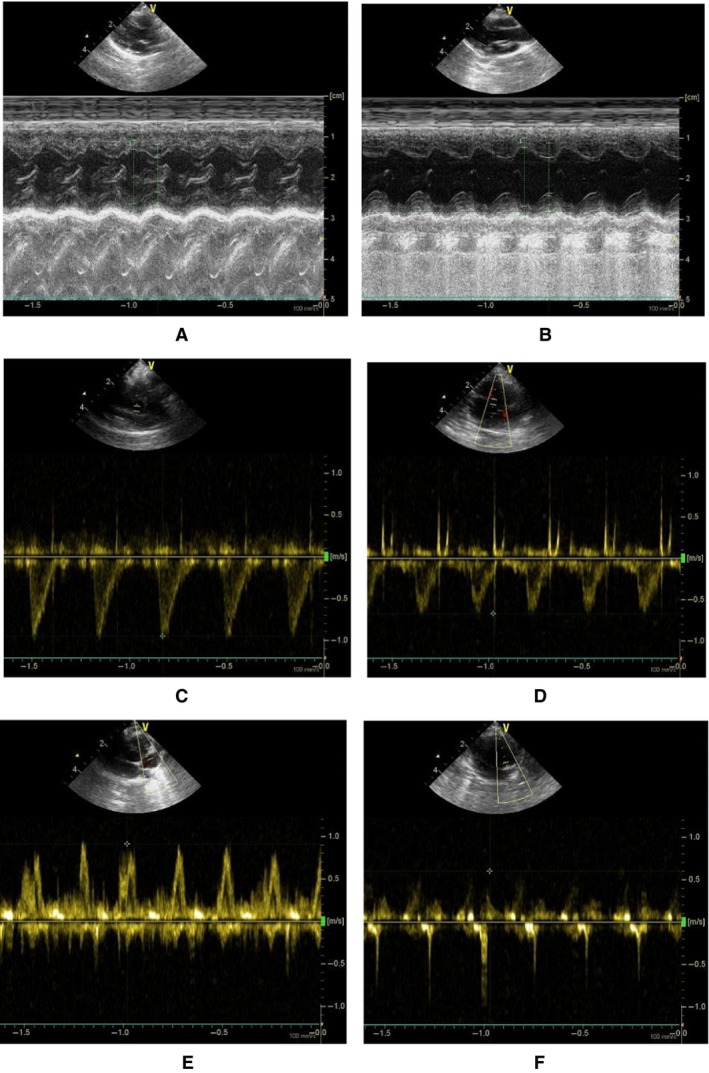
(A–B) Right parasternal short‐axis echocardiographic view with the two‐dimensional guided M‐mode tracing in (A) a sham rabbit and (B) a rabbit of myocardial infarction; (C–D) Subcostal apical five‐chamber echocardiographic view of aortic outflow velocity with the pulsed Doppler tracing in (C) a sham rabbit and (D) a rabbit of myocardial infarction; and (E–F) Subcostal apical four‐chamber echocardiographic view of mitral inflow velocity with the pulsed Doppler tracing in (E) a sham rabbit and (F) a rabbit of myocardial infarction at postoperative 6 weeks.

**Figure 3:**
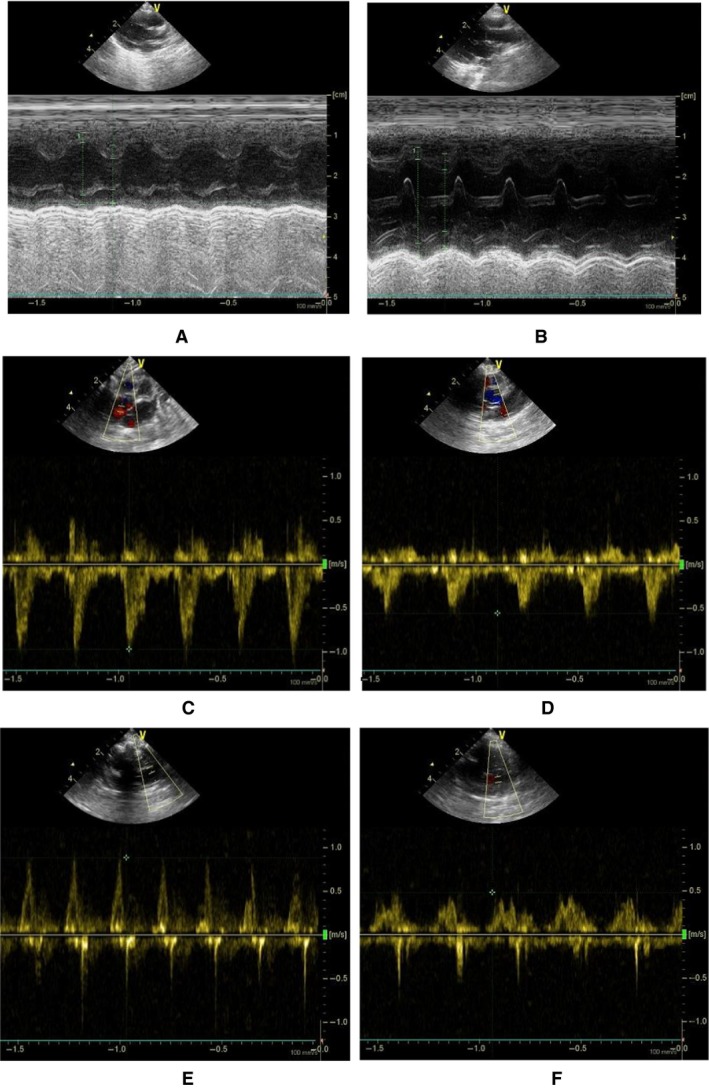
(A–B) Right parasternal short‐axis echocardiographic view with the two‐dimensional guided M‐mode tracing in (A) a sham rabbit and (B) a rabbit of myocardial infarction; (C–D) Subcostal apical five‐chamber echocardiographic view of aortic outflow velocity with the pulsed Doppler tracing in (C) a sham rabbit and (D) a rabbit of myocardial infarction; and (E–F) Subcostal apical four‐chamber echocardiographic view of mitral inflow velocity with the pulsed Doppler tracing in (E) a sham rabbit and (F) a rabbit of myocardial infarction at postoperative 12 weeks.

**Figure 4:**
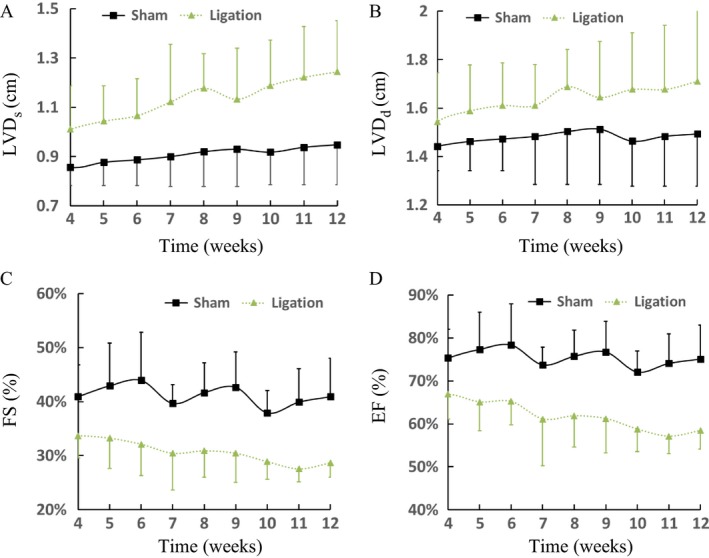
Postoperative changes of (A) LVD_s_, (B) LVD_d_, (C) FS (%), and (D) EF (%) with time. There is statistical difference of the four parameters (*P* < 0.05) between sham and ligation groups.

**Figure 5:**
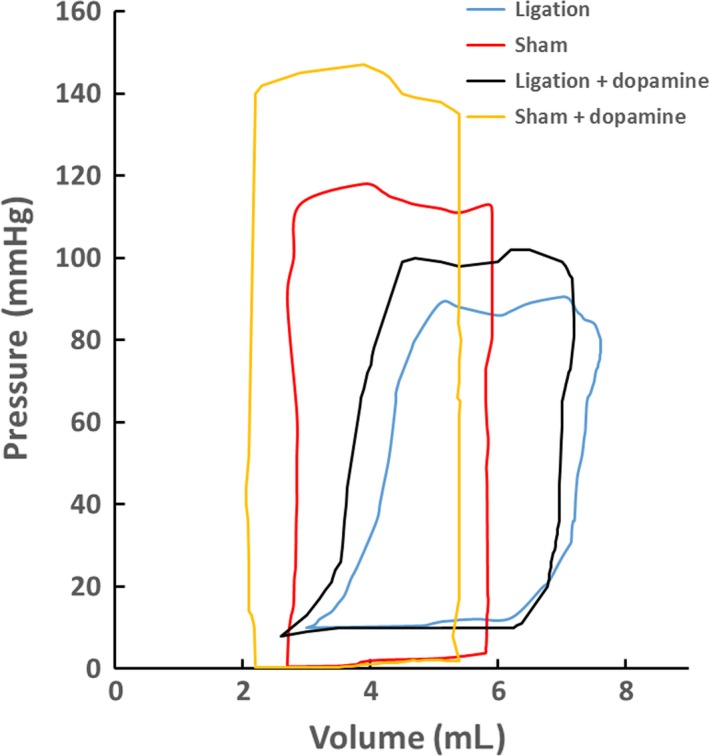
P–V loops in the LV of sham‐operated or post‐MI rabbits with or without dopamine at postoperative 6 weeks.

Figures 2 and 3 show M‐mode measurements of rabbit LV and Doppler measurements of aortic outflow and mitral inflow velocities at postoperative 6 and 12 weeks, respectively. Table 1 lists mean±SD values of IVS_s_, IVS_d_, LVPW_s_, LVPW_d_, aortic sinus diameter (ASD), HR and body weight (BW) between sham and ligation groups for 6 and 12 weeks after the surgery, which have no statistical difference. In contrast, myocardial infraction leads to a significant decrease in APV and MPV (*P* < 0.05). Figure 4 shows the continuous changes of LVD_s_, LVD_d_, FS (%), and EF (%) postoperatively. The difference of four parameters between sham and ligation groups increases with time (*P* < 0.05). Hemodynamic parameters, LVSP and LVEDP, have mean±SD values of 99 ± 20 and 6 ± 3 mmHg in the sham group and 90 ± 16 and 18 ± 11 mmHg in the ligation group at postoperative 12 weeks, which show significant difference of LVEDP (*P* < 0.05). There is a ~40% decrease of $\pm \frac{dp}{dt}$ values in the ligation group as compared with the sham group at postoperative 12 weeks (*P* < 0.05). Moreover, Figure 5 shows P–V loops in the LV of sham‐operated or post‐MI rabbits with or without dopamine. In comparison with the sham group, there is a rightward shift of LV P–V loops as well as a weak response to dopamine in the ligation group.

**Table 1:** Echocardiographic and hemodynamic parameters in sham and ligation groups for 6 and 12 weeks postoperatively

Variable	Sham	Ligation (6 weeks)	Ligation (12 weeks)
IVS_s_ (cm)	0.39 ± 0.12	0.38 ± 0.04	0.33 ± 0.05
IVS_d_(cm)	0.28 ± 0.08	0.27 ± 0.05	0.23 ± 0.05
LVPW_s_ (cm)	0.39 ± 0.11	0.36 ± 0.05	0.34 ± 0.05
LVPW_d_ (cm)	0.27 ± 0.08	0.24 ± 0.05	0.22 ± 0.04
ASD (cm)	0.87 ± 0.08	0.87 ± 0.07	0.87 ± 0.07
APV (m/s)	0.85 ± 0.11	0.66 ± 0.12*	0.60 ± 0.14*
MPV (m/s)	0.75 ± 0.16	0.66 ± 0.11	0.51 ± 0.18*
LVSP	99 ± 20	93 ± 15	90 ± 16
LVEDP	6 ± 3	16 ± 10*	18 ± 11*
$+\frac{dp}{dt}$	5421 ± 578	3894 ± 719*	3326 ± 612*
$-\frac{dp}{dt}$	−4091 ± 772	−2945 ± 553*	−2399 ± 478*
HR (bpm)	207 ± 28	215 ± 29	219 ± 38
BW (Kg)	3.19 ± 0.03	3.02 ± 0.13	2.99 ± 0.16

Superscript * refers to the statistical difference (*P* < 0.05) between sham and ligation groups.

**Figure 6:**
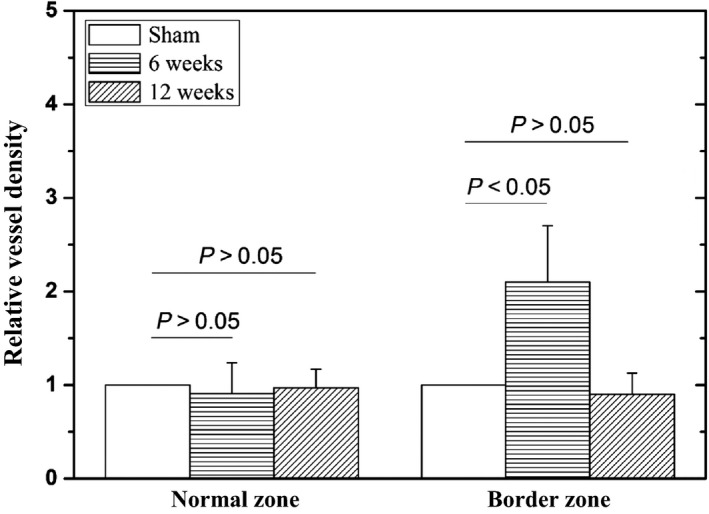
Relative vessel densities (i.e., $\frac{\text{Vessel density}}{\text{Vessel density in sham}}$ and a unit value in the sham group) at normal and border zones of rabbit LV of myocardial infarction at postoperative 6 and 12 weeks, where column and error bars refer to the mean and SD values (averaged over all sectioned plugs, *n* = 5 for each group). This study only computed the density of arterioles with diameter ≥36 *μ*m.

**Figure 7:**
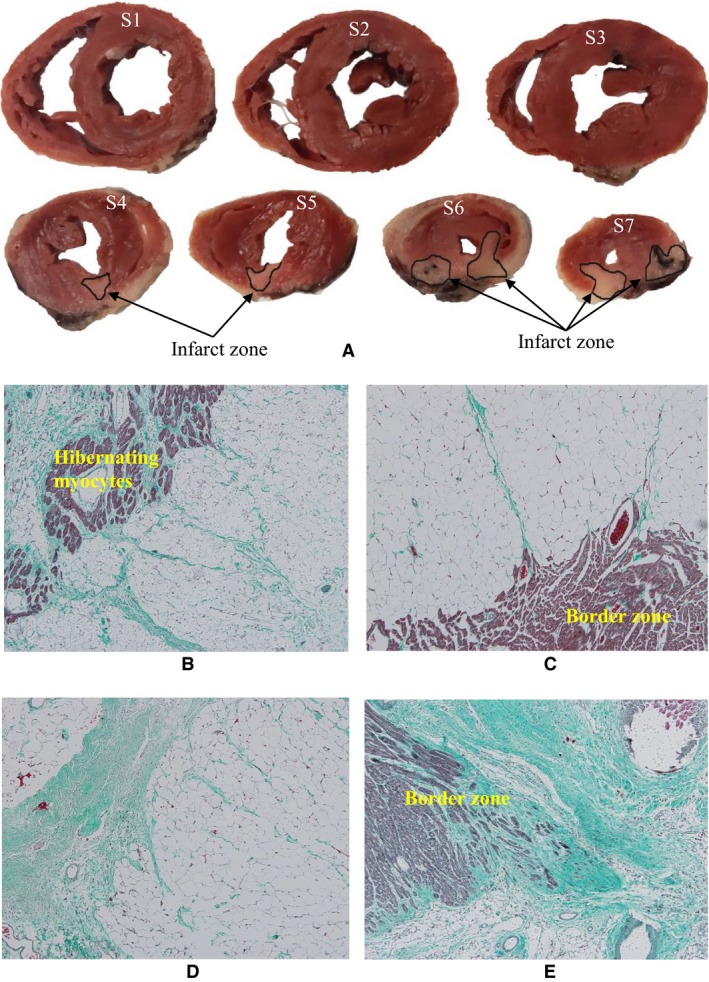
(A) 2,3,5‐Triphenyl‐2H‐tetrazolium chloride (TTC) staining of a rabbit of myocardial infarction at postoperative 6 weeks, where S1‐S7 represent the basal to apical segments of the LV; and (B‐E) Masson trichrome staining of myocardial tissues near the LV apex (i.e., S7 segment) of (B–C) a rabbit of MI at postoperative 6 weeks and (D‐E) a rabbit of MI at postoperative 12 weeks, where Figs. B and D show the infarct zone and Figs. C and E show the border zone. Mahogany and turquoise colors in Figs. B–E refer to myocytes and fibrosis, respectively.

**Figure 8:**
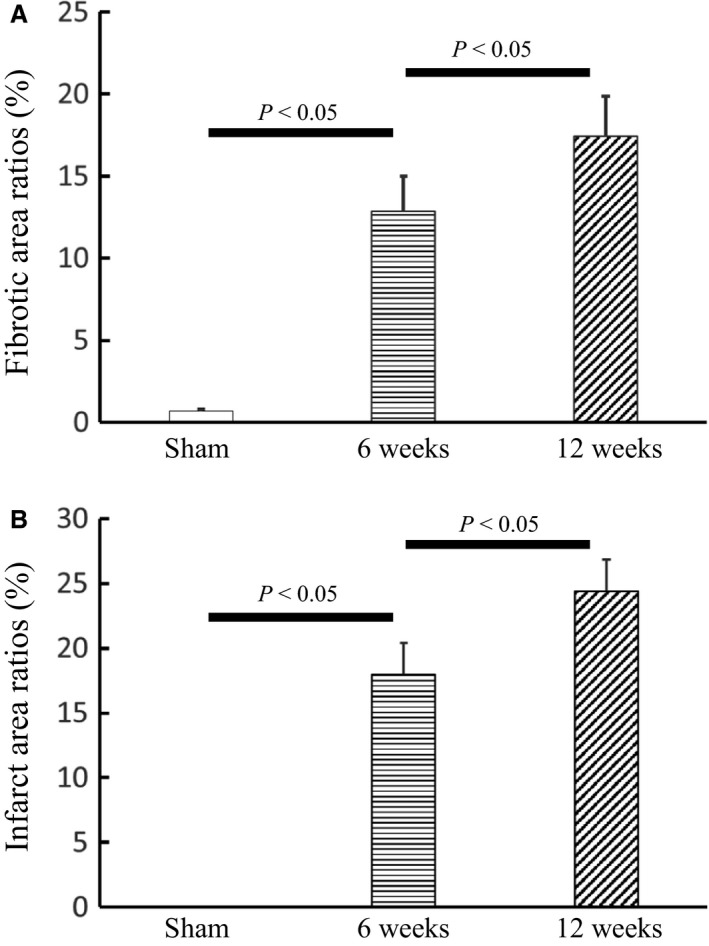
(A) Fibrotic and (B) infarct area ratios (%) of the sham group as well as the ligation group at postoperative 6 and 12 weeks, where column and error bars refer to the mean and SD values (averaged over the corresponding animals, *n* = 5 for each group).

Table 2 lists hematological parameters in sham and ligation groups at postoperative 11 weeks. A significant increase in WBC and NE% indicates strong inflammation while a substantial increase in NRBC% and RDW% shows anoxia and anemia in the ligation group. Based on the *μ*CT measurements, Figure 6 shows mean ± SD values of relative vessel density (i.e., $\frac{\text{Vessel density}}{\text{Vessel density in sham}}$ and a unit value in the sham group) at normal and border zones. Border zones in rabbits of myocardial infarction have a twofold increase in vessel density at postoperative 6 weeks, but a slight decrease at postoperative 12 weeks. There is no statistical difference in normal zones between sham and disease animals. Furthermore, Figure 7A shows infarct zones from the basal to apical segments of the LV. Figures 7B–E show the Masson trichrome staining of myocardial tissues near the LV apex of sham and disease rabbits. Based on the histological measurements, Figure 8 shows a gradual increase in fibrotic and infarct area ratios with time.

**Table 2:** Hematological parameters in sham and ligation groups at postoperative 11 weeks

Variable	Sham	Ligation	*P*
WBC (10^9^ */L)*	7.84 ± 2.60	15.67 ± 5.55	<0.05
RBC (10^12^/*L*)	5.64 ± 0.43	5.52 ± 0.68	0.72
MO (%)	10.52 ± 7.54	16.17 ± 6.50	0.19
E (%)	0.68 ± 0.42	0.35 ± 0.22	0.12
B (%)	0.55 ± 0.26	0.73 ± 0.76	0.58
PDW (%)	15.35 ± 0.62	16.05 ± 1.09	0.20
MPV (fl)	4.87 ± 0.59	5.03 ± 0.25	0.54
PCT (%)	0.19 ± 0.08	0.33 ± 0.11	<0.05
NRBC (%)	0	1.75 ± 2.36	<0.05
HGB (g/*L*)	118.02 ± 7.55	99.83 ± 15.12	<0.05
MCV (fl)	64.38 ± 3.51	54.12 ± 6.25	<0.05
NE (%)	39.18 ± 4.93	56.27 ± 10.27	<0.05
LY (%)	49.08 ± 8.32	26.48 ± 16.32	<0.05
HCT (%)	34.01 ± 6.96	29.82 ± 4.4	0.24
RDW (%)	14.93 ± 1.40	20.0 ± 6.0	0.07

## Discussion

This study developed a post‐MI rabbit model through ligations of the LAD artery and primary branches of the LCx arterial tree. In general, rabbits had a dominant LCx arterial tree, but a thin and small LAD artery (Ziv et al. 2012), as shown in Figure 1. Given the anatomy of coronary arterial trees, the distal LCx ligation was a standard method to induce myocardial infraction at the apical portion of rabbit LV (Mahaffey et al. 1995). To mimic the sequelae of human cardiomyopathy, Schmitto et al. created a reproducible sheep model with ischemic dilation and mitral regurgitation through multiple ligations of coronary arteries on the LV (Schmitto et al. 2010). Here, we performed similar ligations on rabbit LV. There was no surgical mortality except the initial two rabbits for practice. Moreover, three rabbits were dead because of pulmonary congestion at postoperative 4‐6 weeks. The five animals have been excluded from the study. To our knowledge, this is the first study to use multiple coronary artery ligations to investigate the ischemic HF in small animals.

Echocardiographic exams showed an approximately monotonic drop of fractional shortening and ejection fraction with time given a gradual increase in LV diameter in systole and diastole and LV end‐diastolic pressure in the ligation group, but constant values in the sham group. Post‐MI rabbits had the rightward shift of LV P–V loops. These findings indicated the gradually decreased efficiency of LV pumping into the systemic circulation in the ligation group. The persistent enlargement of LV cavity and the decrease in maximum $\pm \frac{dp}{dt}$ values manifested a continuous decrease in contractility during isovolumic contraction, which resulted in a significant decrease in mitral and aortic peak velocities as well as weak mitral regurgitation for 12 weeks postoperatively. There was a slight decrease in BW and septum and posterior wall thickness in systole and diastole with time after the ligation surgery despite no statistical difference between ligation and sham groups. Postmortem exams showed slightly thinning and thickening (no statistical difference) of wall thickness at infarction and border zones, respectively, as compared with normal zones, which cannot be observed through echocardiographic exams. The structure‐function remodeling after multiple coronary artery ligations in rabbit hearts accurately mimics the progression of ischemic HF in post‐MI patients (Cohn et al. 1997; Hunt et al. 2005; Houser et al. 2012; Stevenson et al. 2012; McDiarmid et al. 2017), which validates the proposed rabbit model and supports its application to investigation of ischemic HF in future studies.

A key finding of the study was that compensatory arteriolar growth (i.e., arterioles with diameter ≥36 *μ*m) occurred for 6 weeks after myocardial infarction and converted to the rarefaction from 6 to 12 weeks in border zones, as shown in Figure 6. We have recently found a slight increase in vascular density during the initial period of LV hypertension (Huo and Kassab 2012), but a significant vascular rarefaction in the HF of a swine model (Huo and Kassab 2015). Multiple coronary artery ligations showed the similar vascular remodeling in border zones of rabbit LV. Lee et al. showed much higher wall stress in the border zone in post‐MI patients as compared with the normal zone, given the akinetic infarct region (Lee et al. 2011, 2014, 2016). Here, hemodynamic and echocardiographic measurements showed the elevated end‐diastolic pressure, enlarged LV cavity, and decreased wall thickness, the interplay of which significantly increased the wall stress in the border zone consistent with previous findings (Lee et al. 2011, 2014, 2016). An increase in energy expenditure resulted in compensatory vascular growth to satisfy the enhanced metabolic requirements in border zones during the initial period after MI. A persistent overload, however, led to fatigue and impairment of myocardial tissues and vessels that caused arteriolar rarefaction from 6 to 12 weeks postoperatively (Bayeva et al. 2013).

Another finding was a continuous increase in fibrotic and infarct area ratios after myocardial infarction, as shown in Figure 8. The Masson trichrome staining (Prabhu and Frangogiannis 2016; Talman and Ruskoaho 2016) showed a huge amount of tissues with fibrosis at border zones of rabbit hearts at postoperative 12 weeks given post‐MI inflammation (Anker and von Haehling 2004). An increase in cardiac fibrosis altered the constitutive relation of myocardium and further increased the ventricle wall shear, which impaired coronary vasculature (e.g., a vascular rarefaction) at border zones and contributed to the vicious cycle of ischemia and HF with time.

### A comparison with other animal models

Volume and pressure overload, tachycardia‐pacing, and toxic cardiomyopathy in rabbits are the general methods to evaluate the incidence and progression of congestive heart failure (CHF) (Hasenfuss 1998; Muders and Elsner 2000; Monnet and Chachques 2005; Patten and Hall‐Porter 2009; Houser et al. 2012; Haack et al. 2013). These models are, however, unsuitable for the study of ischemic HF caused by coronary artery diseases. Coronary artery occlusions from the suture ligations, ameroid constrictors, or coiling have been used in mice, rats, rabbits, dogs, and pigs for reproduction of the ischemia‐induced LV dysfunction and HF (Monnet and Chachques 2005; Patten and Hall‐Porter 2009; Dubi and Arbel 2010). In comparison with mouse and rat, the rabbit model allows chronic measurements of hemodynamics, hormonal system, and organ function. Post‐MI rabbits through a distal LCx ligation showed the impaired LV function as well as the activation of neurohormonal and inflammatory systems (Mahaffey et al. 1995; Rungwerth et al. 2004), which presented high similarity to the large animal models of MI (Pfeffer and Braunwald 1990; Moainie et al. 2002). The post‐MI rabbit model is less expensive and easily operated than the large animal models. The present post‐MI rabbit model showed similar structure‐function changes to the model induced by a distal LCx ligation. Furthermore, this model led to myocardial infarction in the anterior wall and apical region of LV and was better to mimic human cardiomyopathy.

### Critique of the study

Although experimental measurements were performed to analyze the structure‐function remodeling after multiple coronary artery ligations in rabbits, computational results of LV wall stresses (Lee et al. 2011, 2014, 2016) are still required to quantitatively analyze the progression of ischemic HF. Fibrotic tissues around arteries deteriorated the vasoactivity (Huo et al. 2012, 2013) and induced the regression of coronary vessels and caused the dearth of hibernating myocytes in the infarct zone, the mechanisms of which should be investigated in relation to the mechanobiology of myocytes (Lyon et al. 2015). Moreover, a comparison of multiple coronary artery ligations with the traditional LCX ligation should be performed in future studies.

## Conclusions

A post‐MI rabbit model was created through multiple ligations of coronary arteries. The in vivo and ex vivo measurements showed the clinically relevant structure‐function remodeling for 12 weeks after MI, for example, persistent enlargement of the LV, slight mitral regurgitation, monotonic drop of fractional shortening and ejection fraction, etc. Moreover, coronary vascular rarefaction and cardiac fibrosis relevant to inflammation occurred concurrently in the border zone at postoperative 12 weeks while there were compensatory vascular growth and scanty fibrosis at postoperative 6 weeks. The interplay of degenerated coronary vasculature and increased LV wall shear, associated with cardiac fibrosis at the border zone, is a risk factor for the vicious cycle of ischemia and HF. The proposed post‐MI rabbit model can serve to investigate basic mechanisms for progression of ischemic HF as well as test anti‐inflammatory agents for treatment.

## Conflict of Interest

None.
